# Supplementary material for: Predicting successful draft outcome in Australian Rules football: Model sensitivity is superior in neural networks when compared to logistic regression
Source: PLoS One. 2024 Feb 29;19(2):e0298743. doi: 10.1371/journal.pone.0298743 (PMC10903873; doi:10.1371/journal.pone.0298743)
Supplement: S1 Table — (DOCX) [file pone.0298743.s001.docx]

| Data | Inclusions |
| --- | --- |
| All Variables | Height (cm), Mass (kg), Reach (cm), Vertical Jump (cm), Running Vertical Jump -R (cm), Running Vertical Jump -R (cm), Running Vertical Jump -L (cm), 5m Speed (m·s^-1^), 10m Speed (m·s^-1^), 20m Speed m·s^-1^), Agility (s), Predicted V̇O_2_ max (ml·kg^-1^·min^-1^), Field Time (min), Total Distance (m), Relative Distance (m·min^-1^), High Speed Efforts, Sprint Efforts, Involvements (n·min^-1^), Disposals (n·min^-1^), Possessions (n·min^-1^), Pressure Acts (n·min^-1^), Positive Involvements (n·min^-1^) |
|  |  |
| Physical Factors |  |
| Anthropometry | Height (cm), Mass (kg), Reach (cm) |
| Speed | 5m Speed (m·s^-1^), 10m Speed (m·s^-1^), 20m Speed (m·s^-1^) |
| Jump | Vertical Jump (cm), Running Vertical Jump -R (cm), Running Vertical Jump -R (cm) |
|  |  |
| GPS Factors |  |
| Running Effort | Relative Distance (m·min^-1^), High Speed Efforts, Sprint Efforts |
| Running Contribution | Field Time (min), Total Distance (m) |
|  |  |
| Technical Involvement | Involvements (n·min^-1^), Disposals (n·min^-1^), Possessions (n·min^-1^), Pressure Acts (n·min^-1^), Positive Involvements (n·min^-1^) |
|  |  |
| Independent | Agility (s), Estimated V̇O_2_ max (ml·kg^-1^·min^-1^) |
